# Survey of Paediatric Oncologists and Pathologists regarding Their Views and Experiences with Variant Translocations in Ewing and Ewing-Like Sarcoma: A Report of the Children's Oncology Group

**DOI:** 10.1155/2020/3498549

**Published:** 2020-12-05

**Authors:** Michael D. Kinnaman, Chong Zhu, Daniel A. Weiser, Sana Mohiuddin, Pooja Hingorani, Michael Roth, Jonathan Gill, Katherine A. Janeway, Richard Gorlick, Stephen L. Lessnick, Patrick J. Grohar

**Affiliations:** ^1^Department of Pediatrics, Memorial Sloan Kettering Cancer Center, New York, NY, USA; ^2^Brii Biosciences, Beijing, China; ^3^Departments of Pediatrics and Genetics, Montefiore Medical Center, Albert Einstein College of Medicine, Bronx, NY, USA; ^4^Department of Pediatrics, University of Texas MD Anderson Cancer Center, Houston, TX, USA; ^5^Department of Pediatric Oncology, Dana-Farber Cancer Institute, Boston, MA, USA; ^6^Center for Childhood Cancer and Blood Diseases at Nationwide Children's Hospital, Division of Pediatric Hematology/Oncology/Blood and Marrow Transplant, The Ohio State University College of Medicine, Columbus, OH, USA; ^7^Division of Oncology, Children's Hospital of Philadelphia, Philadelphia, PA, USA

## Abstract

Advances in molecular diagnostics have identified subsets of Ewing and Ewing-like sarcomas driven by variant translocations with unique biology. It is likely that patients with these tumours will have different clinical features and therapeutic outcomes. Nevertheless, the management of these patients both locally and within cooperative group trials depends on the local pathological diagnosis. It is not known what molecular diagnostic approaches are employed by local pathologists or if the exact translocation is commonly determined. In addition, it is not known what therapeutic approaches are employed for these patients or what cooperative trials are deemed appropriate for these patients by expert consensus. To answer these questions, we performed an international survey of oncologists and pathologists to better understand the diagnostic approaches used to identify variant translocations and the influence the findings have on therapy and clinical trial eligibility. An online survey was distributed to oncologists and pathologists primarily in North America. A total of 141 surveys were completed, representing a 28% response rate. The majority of respondents considered EWSR1-ETS gene family translocations (range 61–96%) to be Ewing sarcoma and would include them on the primary arm of a Ewing sarcoma clinical trial. There was a lack of consensus on how to classify and stratify BCOR-CCNB3, CIC-DUX4, and EWSR1+ with non-ETS partner fusions. Most respondents were either unsure how their institution tested, or their institution did not perform the test. In cases with atypical Ewing morphology, most respondents favoured additional fusion transcript testing. There is a lack of consensus regarding the classification and stratification of rare molecular subtypes in Ewing sarcoma. It is not clear how these alternative translocations have impacted outcomes for past clinical studies. This suggests a need for molecular confirmation of diagnoses and centralized or minimum standardization of testing for future trial enrolment.

## 1. Introduction

Chromosomal rearrangements are the defining molecular feature for a number of sarcomas. Ewing sarcoma is the prototypical example characterized by the t (11; 22) (q24; q12) chromosomal translocation that leads to the expression of the EWSR1-FLI1 transcription factor. The prevalence of EWSR1-FLI1 in Ewing sarcoma is around 85% [1]. The second most common translocation, occurring in 10% of Ewing sarcoma patients, is the t(21; 22) (q22; q12) that generates the EWSR1-ERG transcription factor [1, 2]. In rarer circumstances, EWSR1 is fused to other ETS-family members, including ETV1 (7p22) [3], ETV4 (17q21) [4], and FEV (2q35-36) [5]. EWSR1 is also known to be translocated in 13 other tumour types [6] with at least 17 other fusion partners [6], common partners being, WT1, ATF1, CREB1, YY1, NFATC2, and others [7–22]. In addition, over the last decade, two new histological variants of Ewing sarcoma have been described that result from either a t(4; 19) (q35; q13) or a t(10; 19) (q35; q13) translocation or chromosome X paracentric inversion to generate CIC-DUX4 or BCOR-CCNB3 fusion proteins, respectively [23, 24]. These tumours have likely been included in previous Ewing sarcoma clinical studies at an unknown incidence.

**Figure 1:**
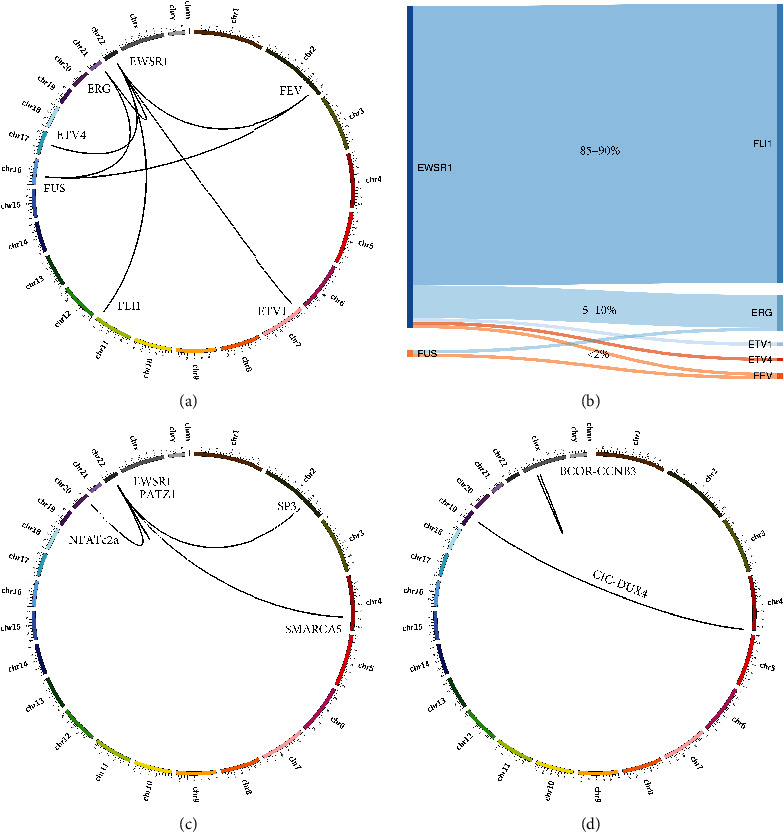
Proposed WHO Classification of Tumours of Soft Tissue and Bone for Ewing and Ewing-like sarcoma: (a) Circos plot of Ewing sarcoma with all FET-ETS variants. (b) Sankey plot of FET-ETS translocation Ewing sarcoma scaled to represent the percentage of total cases. (c) Circos plot of select EWSR1 round cell sarcomas with non-ETS partners. (d) Circos plot of two most common CIC and BCOR translocation sarcomas.

The forthcoming WHO Classification of Tumours of Soft Tissue and Bone will leverage molecular diagnostics and subclassify Ewing and Ewing-like sarcomas into four categories inclusive of genetic translocation: Ewing sarcoma, EWSR1 round cell sarcoma with non-ETS partners, CIC sarcomas, and BCOR sarcomas (Figure 1). Although some studies showed substantial differences in clinical behaviour between tumours with typical Ewing sarcoma translocations and those with rarer rearrangements, the numbers of cases are too small to reach any convincing conclusion [25–28]. Some investigators believe that these patients should be included in Ewing sarcoma clinical trials but analysed separately due to the difficulty in studying the minor subgroups in an already rare disease [29].

Nevertheless, the participation in Ewing sarcoma cooperative group trials has only required a local histologic diagnosis consistent with a Ewing family tumour for enrollment. This implies a need to understand what the consensus approach is to diagnose and treat these variant translocations. It is likely that many sites rely on fluorescence in situ hybridization (FISH) using the EWSR1 gene break-apart probe as the established molecular diagnostic test for Ewing sarcoma. However, as mentioned above, EWSR1 has at least 17 other fusion partners in various soft tissue sarcomas, including EWSR1-WT1 in desmoplastic small round cell tumour (DSCRT), EWSR1-CHOP in myxoid liposarcoma, EWSR1-CHN in extra-skeletal myxoid chondrosarcoma, EWSR1-ATF1 in angiomatoid fibrous histiocytoma, and EWSR1-CREB1 in clear cell sarcoma to name a few [30]. EWSR1 break-apart probes are not able to differentiate these entities from each other and pathologists instead rely on histologic/immunohistochemical findings. Specific FISH rearrangement probes for all the partner genes, RT-PCR, and next-generation sequencing techniques are not universally available, making it difficult to establish a definitive molecular diagnosis in most circumstances.

The goal of this study is to better understand oncologists and pathologists' views on the classifications and management of variant chromosomal rearrangements previously reported in small round cell tumours resembling Ewing sarcoma. We seek to understand which tests are most commonly used in clinical practice and how these findings may inform inclusion and stratification of molecular subgroups for future Ewing sarcoma clinical trials. Herein, we performed an international survey of oncologists and pathologists' regarding their current approach to classification and identification of variant translocations and how they would stratify these rare variant translocations on future Ewing sarcoma clinical trials.

## 2. Materials and Methods

A cross-sectional study with a descriptive design was used. The focus of the survey was to investigate paediatric oncologists' and pathologists' views and experiences with chromosomal rearrangements relating to Ewing sarcoma and to describe the landscape of chromosomal translocations in Ewing sarcoma, Ewing-like sarcoma, and other sarcomas harbouring the EWSR1 translocation.

### 2.1. Survey Design and Implementation

The survey items were developed with a desire to obtain providers' opinions on the designation and eligibility to be enrolled on a Ewing sarcoma clinical trial based on a list of variant translocations that have been reported in Ewing sarcoma. The survey instrument was created by research fellows and paediatric oncologists at the Children's Hospital at Montefiore. The questionnaire was piloted by oncologists and pathologists specializing in soft tissue sarcoma with revisions for readability. Once the final questionnaire was approved, the e-mail addresses of practicing paediatric oncologists and pathologists were obtained via hospital websites and published manuscripts. Paediatric oncologists were e-mailed a link to an anonymous survey on http://surveymonkey.com. The paediatric oncologists were sent two subsequent reminder e-mails to maximize the response rate. The research protocol and survey were approved by the Institutional Review Board at the Albert Einstein College of Medicine.

Information was ascertained about oncologists' and pathologists' demographics, including their specific discipline, current location of practice, academic affiliation, training, patient volume, practice characteristics, and time spent on basic science research. Respondents were then presented with a clinical vignette and were asked to classify and characterize fourteen different chromosomal translocations as either classic Ewing sarcoma requiring Ewing sarcoma therapy, nonclassic Ewing sarcoma requiring Ewing sarcoma therapy, nonclassic Ewing sarcoma requiring soft tissue sarcoma treatment, or unsure. Respondents were then questioned on whether they would consider the specific fusion type eligible for a Ewing sarcoma clinical trial and which diagnostic tests were available at their institution to identify the proposed translocation. Finally, respondents were questioned regarding when they would find it necessary to perform FISH or further molecular testing when presented with a case of typical Ewing sarcoma morphology and CD 99 positive verse a case with atypical Ewing sarcoma morphology or CD 99 negative staining.

### 2.2. Statistical Analysis

The primary objective of this study was how paediatric oncologists and pathologists would classify variant chromosomal rearrangement previously reported in small round cell tumours resembling Ewing sarcoma histology. The survey was sent to approximately 500 oncologists and pathologists. Based on previous survey protocols, we expected a response rate of approximately 20%. Descriptive data were collected, including frequency, mean, standard deviation, median, range, and quadrants. Results are described using descriptive statistics.

## 3. Results

A total of 141 surveys were completed, representing a 28% response rate. Of those who responded, 78% were paediatric or medical oncologist and 22% were surgical pathologists. Fifty-eight percent of paediatric or medical oncologists and 96% of surgical pathologists indicated that they had a special interest in sarcomas. Most respondents were from academic (90%) practices located in North America (94%) and indicated that they had between 6 and 10 (31%) or >15 (33%) years of experience in the field after completion of training. When asked to describe the number of newly diagnosed bone and soft tissue sarcoma patients they treated each year, most respondents replied between 11 and 25 (31%) or 0 and 10 (25%). Just less than half of respondents (46%) indicated that they spend no time on basic science research (see Table 1 for respondent demographics).

**Figure 2:**
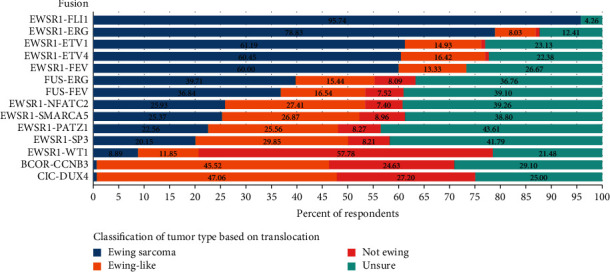
Classification of variant chromosomal translocations by percentage of respondents. The data represent the percentage of respondents who believed a given translocation in the column on the left represents Ewing sarcoma (blue bar and percentage), Ewing-like sarcoma (orange bar and percentage), not Ewing sarcoma (red bar and percentage), or unsure of the classification (teal bar and percentage).

Descriptive statistics depicted in Figure 2 show a heterogeneous range of responses regarding the classification of fourteen different chromosomal translocations in a patient with small round blue cell sarcoma found to be diffusely CD99 positive with membranous staining. There was near consensus regarding the classification of the canonical EWSR1-FLI1 translocation with greater than 95% of respondents classifying this as Ewing sarcoma. A majority of respondents considered EWSR1-ERG, EWSR1-FEV, EWSR1-ETV4, and EWSR1-ETV1 to be Ewing sarcoma. Respondents generally favoured FUS-ERG and FUS-FEV (40% and 37%, respectively) to be Ewing sarcoma; however, almost an equal number of respondents were unsure of how to classify these fusions (37% and 39%, respectively). There was consensus amongst respondents that both BCOR-CCNB3 and CIC-DUX4 translocations were not Ewing sarcoma; however, there was disagreement on whether they should be considered Ewing-like sarcoma or not Ewing sarcoma with almost half of the respondents classifying these both as Ewing-like sarcoma and about one-quarter of respondents classifying these translocations as either not Ewing sarcoma or unsure how to classify them. About 40% of respondents were unsure of how to classify EWSR1-NFATC2, EWSR1-SMARCA5, EWSR1-PATZ1, and EWSR1-SP3. Those who did classify them were split evenly between Ewing sarcoma and Ewing-like sarcoma. Finally, a majority of respondents (58%) recognized the EWSR1-WT1 fusion protein as indicative of a tumour that is not Ewing sarcoma.

**Figure 3:**
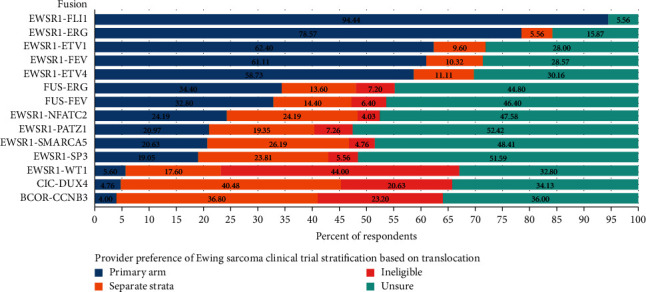
Perceptions of respondents on the optimal stratification of variant chromosomal translocations on future Ewing sarcoma clinical trials. The data represent the percentage of respondents who believed that a given translocation in the column on the left should result in inclusion in a Ewing sarcoma clinical trial in the primary stratum (blue bar and percentage), in a separate stratum (orange bar and percentage) or if the translocation makes a patient ineligible (red bar and percentage) for a Ewing sarcoma study. The teal bar and percentage indicate the number of respondents who were unsure of how the translocation should influence inclusion criteria.

The data showed a similar lack of consensus as to how the presence of a given translocation should influence inclusion in a Ewing sarcoma clinical trial (Figure 3). Not surprisingly, again there was near consensus regarding including EWSR1-FLI1 patients on the primary arm. The only other translocations where a majority of respondents would include on the primary arm of a Ewing sarcoma clinical trial were EWSR1-ERG, EWSR1-ETV1, EWSR1-ETV4, and EWSR1-FEV. Almost half of the respondents were unsure how they would classify patients with FUS-FEV, FUS-ERG, EWSR1-PATZ1, EWSR1-SP3, EWSR1-SMARCA5, and EWSR1-NFATC2. There was not a clear consensus on how BCOR-CCNB3 and CIC-DUX4 translocations should be classified, with a plurality of responses favouring to include these patients on a separate stratum; however, most respondents felt that these should either be ineligible or were not sure how to classify these translocations.

**Figure 4:**
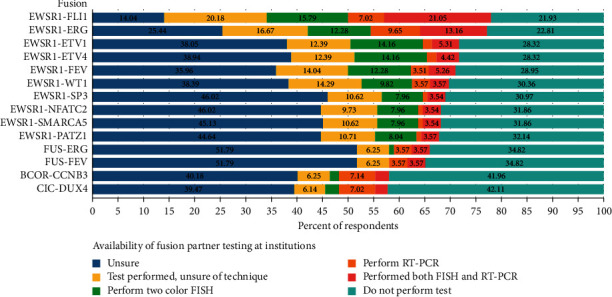
Available variant translocation fusion partner testing at respondent institutions. Data represent the respondent's impression of the type of testing that is done at the local institution to determine fusion partner identity by two-color fish (green bar and percentage), RT-PCR (orange bar and percentage), or both (green bar and percentage). Other investigators were either unsure if testing is performed (blue bar and percentage), unsure of the type of testing (yellow bar and percentage), or do not perform testing (green bar and percentage).

The availability of fusion partner testing at respondent institutions varied by type of fusion (Figure 4). Most fusions' respondents were either unsure of how they tested for the particular fusion or their institution did not perform the test. For the EWSR1-FLI1 fusion, less than half of respondents were able to identify the specific testing used to identify the fusion with slightly more institutions favouring performing both FISH and RT-PCR to two color FISH alone.

**Figure 5:**
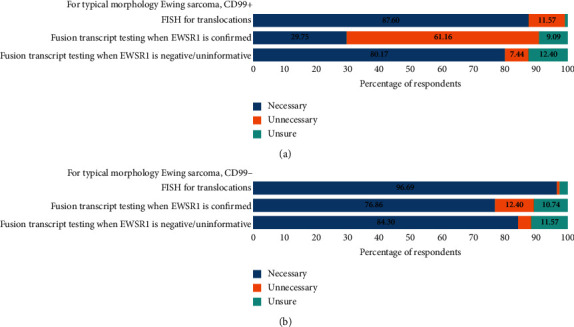
Summary of suggested workup for patients with defined diagnostic features: (a) respondents' opinions regarding the necessary diagnostic workup for a case of a CD99+, typical Ewing morphology tumour. (b) Respondents' opinions regarding the necessary diagnostic workup for a case of a CD99−, atypical Ewing morphology tumour.

For a patient with typical morphology, CD99 positive Ewing sarcoma, 88% of respondents felt FISH was a necessity to confirm the diagnoses (Figure 5). If EWSR1 break-apart was confirmed by FISH, a majority of respondents (61%) felt that it was unnecessary to identify the specific fusion partners. If the EWSR1 break-apart was negative, most respondents (80%) felt that further testing for specific fusion transcripts was necessary.

In a patient with atypical morphology or CD99 negative Ewing sarcoma, almost all respondents (97%) felt that FISH was necessary to confirm the diagnoses of Ewing sarcoma (Figure 5). A majority of respondents (77%) also felt that it was necessary for additional testing for the specific fusion transcript if EWSR1 was confirmed by FISH. In patients where EWSR1 break-apart was negative or uninformative, a majority (84%) of respondents felt that additional testing for specific fusion transcripts was warranted.

## 4. Discussion

Novel molecular alterations in patients presenting with tumours that histologically represent Ewing sarcoma, but lack the canonical EWSR1-ETS gene family translocation, have challenged how we approach diagnosis and classification of these tumours. In the forthcoming fifth edition of the WHO Classification of Tumours of Soft Tissue and Bone, it has been proposed to subclassify Ewing and Ewing-like sarcoma into four categories inclusive of genetic translocation: Ewing sarcoma, EWSR1 round cell sarcoma with non-ETS partners, CIC sarcomas, and BCOR sarcomas. With this new understanding of Ewing sarcoma biology, there is not a clear consensus on whether a molecular characterization of the specific fusion transcript should be required for diagnosis and stratification on future Ewing sarcoma clinical trials. Our survey study provides several key insights into how oncologists and pathologists view the landscape of chromosomal rearrangements relating to Ewing sarcoma and other EWSR1 translocations, their experiences with different testing methodologies, and how they would classify these novel fusions in future clinical trials.

This survey of 141 pathologists and oncologists revealed that while there was a consensus that tumours with EWSR1-ETS gene family translocations should be considered Ewing sarcoma, there is a lack of consensus regarding how to classify rare Ewing sarcoma variants, *EWSR1*+ with non-ETS partner sarcomas, and both CIC and BCOR sarcomas. Several reasons may explain the lack of consensus regarding the classification of these fusions. These are rare fusions in a rare cancer, and combined account for less than 2% of all reported cases [2, 3, 5, 23, 24, 29, 31–39]. In addition, only 42% of our respondents identified as having a special interest in sarcoma. For example, FUS-ERG and FUS-FEV fusions are typically classified as Ewing sarcoma; however, respondents felt much less confident about classifying this as Ewing sarcoma in comparison to other rare EWSR1-FLI1-related gene variants. FU*S* is highly related to EWSR1, and both are members of the FET (FUS/TLS, EWS, and TAF15) family of RNA binding proteins, while FLI1 and its related genes are members of the ETS family of DNA binding proteins, and together the “FET-ETS” fusions represent nearly all cases of Ewing sarcoma. FUS, which stands for “fused in sarcoma,” is used interchangeably with TLS, or “translated in sarcoma,” which may have created some confusion amongst our respondents in being familiar with these fusions. Many respondents may also not be familiar with the FUS subset because their institution may only use EWSR1 break-apart FISH testing for diagnosis, which would miss these fusions all together.

The group of fusions classified as EWSR1 round cell sarcoma with non-ETS partners (EWSR1-NFATC2, EWSR1-SMARCA5, EWSR1-PATZ1, and EWSR1-SP3) was a difficult group for the respondents to classify. Removing those who were unsure how to classify these fusions, respondents were split on whether to classify these as Ewing sarcoma and Ewing-like sarcoma. This confusion is understandable as these fusions represent the rarest variants as they have been only reported in small case series or case reports [29, 32–35]. It is not known how these tumours should be classified and whether they respond to Ewing sarcoma therapies. DNA methylation profiling of an EWSR1-NFATC2 fusion-positive tumour revealed a pattern that segregated out in a homogenous cluster distinct from Ewing sarcoma samples with EWS-ETS translocations, providing evidence that this subclass has unique biology [40]. Further molecular profiling and methylation studies would help understand this unique group of fusions.

About half of the survey respondents favoured labelling BCOR-CCNB3 and CIC-DUX4 fusion-positive tumours as Ewing-like sarcoma, while the other half either were not sure or favoured labelling these as not Ewing sarcoma. Similar to the EWSR1 round cell sarcoma with non-ETS partners subset of fusions, this may be because CIC and BCOR sarcomas are rare and would not be picked up using morphology, immunohistochemistry, or EWSR1 break-apart FISH. BCOR-CCNB3 and CIC-DUX4 fusion-positive tumours represent a group of small round blue cell tumours that are histologically similar to Ewing sarcoma but do not have EWSR1 gene rearrangements. The BCOR sarcomas are characterized by paracentric inversion of chromosome X, resulting in the creation of a BCOR-CCNB3 inv(*x*) (p11; p11) fusion gene [24]. Alternative BCOR partners have been reported including BCOR-MAML3 t(4; *x*) (p11; q31) and ZC3H7-BCOR t(*x*; 22) (p11; q13.2) [41]. Survival for patients with BCOR-CCNB3 rearrangements is similar to Ewing sarcoma, with reported 5-year survival rates of 72% and 76.5% in two small cases series [25, 42]. The CIC-DUX4 t(4; 19) or t(10; 19) translocation is the most common fusion reported in patients with EWSR1/FUS negative small round blue cell tumours [43]. Patients with tumours harbouring this fusion have a particularly aggressive disease course, with a 5-year overall survival of 43%, compared to 77% in a matched Ewing sarcoma group [43]. While it is clear that those with BCOR-CCNB3 fusions benefit from Ewing-type therapy, patients with CIC-DUX4 fusions have worse outcomes and may benefit from alternative treatment approaches.

In this survey, there was not a clear consensus regarding how to stratify and which fusions to include on a future Ewing sarcoma clinical trial. There was general agreement that EWSR1-ETS gene family fusions should be included on the primary arm of a future Ewing sarcoma clinical trial. Respondents were mostly unsure of how to classify FUS-ETS gene family fusions and EWSR1 round cell sarcoma with non-ETS partners, which is consistent with their difficulty classifying these tumours and explained by their rare presentation. There was no clear consensus on how to stratify CIC-DUX4 or BCOR-CCNB3 tumours. While a plurality felt they should be included on a separate stratum, about 20% for each subtype felt that these patients should not be included. As both of these subtypes can be successfully treated with Ewing-type therapy, there is a strong argument to be made for future inclusion on Ewing sarcoma clinical trials.

Advances in molecular diagnostics have demonstrated that the Ewing sarcoma family of tumours represents a heterogeneous group of tumours that are defined by their chromosomal translocation. In a review of 200 small round cell tumours where EWSR1 FISH analysis was non-informative, RT-PCR was used to identify rare variant fusions. A majority (66.5%) had EWSR1-ETS gene family translocation identified, while CIC-DUX4, BCOR-CCNB3, SYT-SSX, and EWSR1-WT1 translocations were identified in a smaller subset (0.5–3%) [44], highlighting that many of these rare translocations may be missed without advanced molecular testing. Many of our respondents indicated that either they were unaware of what testing was available or their institution did not perform the testing needed to identify these rare variant translocations. Break-apart FISH probes for the EWSR1 gene are inadequate to establish a molecular diagnosis and will miss FUS translocations as there is no EWSR1 gene that is translocated. In a study of 85 small blue round cell tumours, 8.2% of cases harboured a FUS gene rearrangement and break-apart FISH missed an additional 4 EWSR1-ERG translocations, highlighting the limitations of this approach [45]. Break-apart FISH may falsely classify a small round tumour with a fusion of a different ETS-family member with EWSR1, such as DSRCT or clear cell sarcoma, as Ewing sarcoma.

The most recent Children's Oncology Group Ewing sarcoma phase 3 clinical trial (AEWS1221), which was activated in December 2014, required a local histologic diagnosis consistent with a Ewing family tumour for eligibility in the study. In translocation-positive sarcomas, the consensus is that the biology of the tumour is defined by the underlying translocation and resulting fusion protein. This implies that the fusion protein contributes in a large way to response to therapy. It would, therefore, seem that accruing patients by fusion type make sense. Indeed, in many ways, this survey supports this idea as the majority of respondents felt that patients with the various EWSR1-ETS fusions should accrue on Ewing sarcoma clinical studies. In contrast, only a minority of respondents felt that patients with alternative fusions should accrue in the primary arm with respondents divided among ineligible, and accrue in separate strata or unsure. Nevertheless, it is quite clear from this survey that there is not a uniform approach to molecular testing in these patients with the caveat that only 25% of our respondents were pathologists. Therefore, this study highlights the need for centralized testing, or at minimum, a uniform approach to testing before enrolment on future cooperative studies.

The limitations of the present study include the high percentage of nonrespondents which could lead to a selection bias amongst respondents. It is possible that only those oncologists and pathologists who already had an interest in this topic would respond. The implications of this bias are that the inferences that can be drawn from this study are limited. We are not able to provide a comprehensive assessment from all providers who provide care to patients with Ewing and Ewing-like sarcoma and the diagnostic tools that are available to them. Our study is also regionally biased, as 90% of respondents were from North America, making the generalizability of our findings to institutions outside of North America, especially those in resource-limited settings, challenging. Finally, we did not include differences in the break-apart region on this survey as we felt that these nuances would be difficult to represent. However, differences in break-apart have also been associated with differences in responses to current treatment regimens.

## 5. Conclusions

In conclusion, our study demonstrates that there is a clear lack of consensus regarding which molecular subtypes should be classified and included on future Ewing sarcoma clinical trials. Most respondents believe that EWSR1-ETS fusions should accrue in the primary arm of Ewing studies while there is no consensus on eligibility for alternative fusions. There is also a lack of consistency regarding diagnostic workup across institutions. It is not clear how these alternative fusions have impacted reported outcomes for past clinical studies in Ewing sarcoma. These findings are consistent with the need for centralized testing and/or a uniform approach to molecular testing for patients enrolled on future Ewing sarcoma clinical trials particularly in light of the forthcoming update to the WHO Classification of Bone and Soft Tissue Tumours.

## Figures and Tables

**Table 1:** Demographics of respondents.

Demographics	Count of respondents (%)
*Discipline*	
Pediatric/medical oncologists	85 (78.0)
W/ special interest in sarcomas	49 (57.6)
W/o special interest in sarcomas	36 (42.4)
Surgical pathologists	24 (22.0)
W/ special interest in sarcomas	23 (95.8)
W/o special interest in sarcomas	1 (4.2)
Sarcoma specialists	72 (66.1)
Nonsarcoma specialists	37 (33.9)

*Current location of practise*	
North America	102 (93.6)
Europe	3 (2.8)
Asia and Pacific	4 (3.7)

*Type of practice*	
Academic	98 (89.9)
Private/community	11 (10.1)

*Duration after completion of training (years)*	
1–5	25 (22.9)
6–10	34 (31.2)
11–15	14 (12.8)
>15	36 (33.0)

*Number of newly diagnosed bone and soft tissue sarcoma patients treated each year*	
0–10	27 (24.8)
11–25	34 (31.2)
26–50	19 (17.4)
51–100	8 (7.3)
>100	21 (19.3)

*Percentage of time spent on basic science research*	
0	50 (45.9)
1–20%	22 (20.2)
21–60%	19 (17.4)
61–100%	18 (16.5)

## Data Availability

The data used to support the findings of this study are available from the corresponding author upon request.

## Supplementary Materials

Supplementary Materials Ewing translocation survey results.
