# Supplementary material for: Survey of Paediatric Oncologists and Pathologists regarding Their Views and Experiences with Variant Translocations in Ewing and Ewing-Like Sarcoma: A Report of the Children's Oncology Group
Source: Sarcoma. 2020 Dec 5;2020:3498549. doi: 10.1155/2020/3498549 (PMC7787769; doi:10.1155/2020/3498549)
Supplement: Supplementary Materials — Ewing translocation survey results. [file 3498549.f1.pdf]

Supplementary Materials - Ewing Translocation Survey Results

**Q1: You are seeing a patient who presented with a mass in the right lower extremity. Biopsy was performed. Histology demonstrated a small round blue cell sarcoma and immunohistochemical staining showed diffuse CD99 positive staining. Molecular tests were performed and variant**
**fusion transcripts were confirmed. With each of the following variants, how do you classify the case? (Please note EWSR1 = EWS in the following text.)**
*Answered: 141 Skipped: 2*

| | EWING SARCOMA | "EWING-LIKE" SARCOMA | NOT A EWING SARCOMA | UNSURE | TOTAL |
|---|---|---|---|---|---|
| EWSR1-FLI1 | 95.74%<br>135 | 0.00%<br>0 | 0.00%<br>0 | 4.26%<br>6 | 141 |
| EWSR1-ERG | 78.83%<br>108 | 8.03%<br>11 | 0.73%<br>1 | 12.41%<br>17 | 137 |
| EWSR1-FEV | 60.00%<br>81 | 13.33%<br>18 | 0.00%<br>0 | 26.67%<br>36 | 135 |
| EWSR1-ETV4(E1AF) | 60.45%<br>81 | 16.42%<br>22 | 0.75%<br>1 | 22.39%<br>30 | 134 |
| EWSR1-ETV1 | 61.19%<br>82 | 14.93%<br>20 | 0.75%<br>1 | 23.13%<br>31 | 134 |
| FUS-ERG | 39.71%<br>54 | 15.44%<br>21 | 8.09%<br>11 | 36.76%<br>50 | 136 |
| FUS-FEV | 36.84%<br>49 | 16.54%<br>22 | 7.52%<br>10 | 39.10%<br>52 | 133 |
| EWSR1-NFATC2 | 25.93%<br>35 | 27.41%<br>37 | 7.41%<br>10 | 39.26%<br>53 | 135 |
| EWSR1-SMARCA5 | 25.37%<br>34 | 26.87%<br>36 | 8.96%<br>12 | 38.81%<br>52 | 134 |
| EWSR1-SP3 | 20.15%<br>27 | 29.85%<br>40 | 8.21%<br>11 | 41.79%<br>56 | 134 |
| EWSR1-PATZ1(ZSG) | 22.56%<br>30 | 25.56%<br>34 | 8.27%<br>11 | 43.61%<br>58 | 133 |
| CIC-DUX4 | 0.74%<br>1 | 47.06%<br>64 | 27.21%<br>37 | 25.00%<br>34 | 136 |
| BCOR-CCNB3 | 0.75%<br>1 | 45.52%<br>61 | 24.63%<br>33 | 29.10%<br>39 | 134 |
| EWSR1-WT1 | 8.89%<br>12 | 11.85%<br>16 | 57.78%<br>78 | 21.48%<br>29 | 135 |

**Q2: Would patients with the following tumors get Ewing therapy?**
*Answered: 140 Skipped: 3*

| | YES | NO | UNSURE | TOTAL |
|---|---|---|---|---|
| Ewing sarcoma | 99.29%<br>139 | 0.00%<br>0 | 0.71%<br>1 | 140 |
| Ewing-like sarcoma | 90.58%<br>125 | 1.45%<br>2 | 7.97%<br>11 | 138 |
| Desmoplastic small round cell tumor | 38.41%<br>53 | 39.13%<br>54 | 22.46%<br>31 | 138 |

## Q3: Would you consider the patient with each of the following fusions eligible for a Ewing sarcoma clinical trial?

*Answered: 126 Skipped: 17*

| | EWING TRIAL- PRIMARY ARM | EWING TRIAL- SEPARATE STRATA | EWING TRIAL- INELIGIBLE | UNSURE | TOTAL |
|---|---|---|---|---|---|
| EWSR1-FLI1 | 94.44% 119 | 0.00% 0 | 0.00% 0 | 5.56% 7 | 126 |
| EWSR1-ERG | 78.57% 99 | 5.56% 7 | 0.00% 0 | 15.87% 20 | 126 |
| EWSR1-FEV | 61.11% 77 | 10.32% 13 | 0.00% 0 | 28.57% 36 | 126 |
| EWSR1-ETV4(E1AF) | 58.73% 74 | 11.11% 14 | 0.00% 0 | 30.16% 38 | 126 |
| EWSR1-ETV1 | 62.40% 78 | 9.60% 12 | 0.00% 0 | 28.00% 35 | 125 |
| FUS-ERG | 34.40% 43 | 13.60% 17 | 7.20% 9 | 44.80% 56 | 125 |
| FUS-FEV | 32.80% 41 | 14.40% 18 | 6.40% 8 | 46.40% 58 | 125 |
| EWSR1-NFATC2 | 24.19% 30 | 24.19% 30 | 4.03% 5 | 47.58% 59 | 124 |
| EWSR1-SMARCA5 | 20.63% 26 | 26.19% 33 | 4.76% 6 | 48.41% 61 | 126 |
| EWSR1-SP3 | 19.05% 24 | 23.81% 30 | 5.56% 7 | 51.59% 65 | 126 |
| EWSR1-PATZ1(ZSG) | 20.97% 26 | 19.35% 24 | 7.26% 9 | 52.42% 65 | 124 |
| CIC-DUX4 | 4.76% 6 | 40.48% 51 | 20.63% 26 | 34.13% 43 | 126 |
| BCOR-CCNB3 | 4.00% 5 | 36.80% 46 | 23.20% 29 | 36.00% 45 | 125 |
| EWSR1-WT1 | 5.60% 7 | 17.60% 22 | 44.00% 55 | 32.80% 41 | 125 |

## Q4: Are break apart FISH tests for the following genes performed at your institution?

*Answered: 121 Skipped: 22*

| | YES | NO | UNSURE | TOTAL |
|---|---|---|---|---|
| EWSR1 | 82.64% 100 | 9.92% 12 | 7.44% 9 | 121 |
| FUS | 36.67% 44 | 30.83% 37 | 32.50% 39 | 120 |
| CIC | 16.53% 20 | 47.93% 58 | 35.54% 43 | 121 |
| BCOR | 14.88% 18 | 49.59% 60 | 35.54% 43 | 121 |

**Q5: Please select the techniques for all tests that are currently performed at your institution to detect fusion gene status.**
*Answered: 114 Skipped: 29*

| | PERFORM TEST, UNSURE OF TECHNIQUE | PERFORM TWO COLOR FISH (FOR SPECIFIC FUSION) | PERFORM RT-PCR | PERFORM BOTH FISH AND RT-PCR | DO NOT PERFORM TEST | UNSURE OF WHETHER TEST IS PERFORMED OR NOT | TOTAL |
|---|---|---|---|---|---|---|---|
| EWSR1-FLI1 | 20.18% 23 | 15.79% 18 | 7.02% 8 | 21.05% 24 | 21.93% 25 | 14.04% 16 | 114 |
| EWSR1-ERG | 16.67% 19 | 12.28% 14 | 9.65% 11 | 13.16% 15 | 22.81% 26 | 25.44% 29 | 114 |
| EWSR1-FEV | 14.04% 16 | 12.28% 14 | 3.51% 4 | 5.26% 6 | 28.95% 33 | 35.96% 41 | 114 |
| EWSR1-ETV4(E1AF) | 12.39% 14 | 14.16% 16 | 1.77% 2 | 4.42% 5 | 28.32% 32 | 38.94% 44 | 113 |
| EWSR1-ETV1 | 12.39% 14 | 14.16% 16 | 1.77% 2 | 5.31% 6 | 28.32% 32 | 38.05% 43 | 113 |
| EWSR1-NFATC2 | 9.73% 11 | 7.96% 9 | 0.88% 1 | 3.54% 4 | 31.86% 36 | 46.02% 52 | 113 |
| EWSR1-SMARCA5 | 10.62% 12 | 7.96% 9 | 0.88% 1 | 3.54% 4 | 31.86% 36 | 45.13% 51 | 113 |
| EWSR1-SP3 | 10.62% 12 | 7.96% 9 | 0.88% 1 | 3.54% 4 | 30.97% 35 | 46.02% 52 | 113 |
| EWSR1-PATZ1 | 10.71% 12 | 8.04% 9 | 0.89% 1 | 3.57% 4 | 32.14% 36 | 44.64% 50 | 112 |
| EWSR1-WT1 | 14.29% 16 | 9.82% 11 | 3.57% 4 | 3.57% 4 | 30.36% 34 | 38.39% 43 | 112 |
| FUS-ERG | 6.25% 7 | 0.89% 1 | 3.57% 4 | 3.57% 4 | 34.82% 39 | 50.89% 57 | 112 |
| FUS-FEV | 6.25% 7 | 0.00% 0 | 3.57% 4 | 3.57% 4 | 34.82% 39 | 51.79% 58 | 112 |
| CIC-DUX4 | 6.14% 7 | 2.63% 3 | 7.02% 8 | 2.63% 3 | 42.11% 48 | 39.47% 45 | 114 |
| BCOR-CCNB3 | 6.25% 7 | 1.79% 2 | 7.14% 8 | 2.68% 3 | 41.96% 47 | 40.18% 45 | 112 |

**Q6: Would you consider it necessary to request FISH testing for translocations for the diagnosis of Ewing sarcoma?**
*Answered: 121 Skipped: 22*

| | YES | NO | UNSURE | TOTAL |
|---|---|---|---|---|
| EWSR1 | 82.64% 100 | 9.92% 12 | 7.44% 9 | 121 |
| FUS | 36.67% 44 | 30.83% 37 | 32.50% 39 | 120 |
| CIC | 16.53% 20 | 47.93% 58 | 35.54% 43 | 121 |
| BCOR | 14.88% 18 | 49.59% 60 | 35.54% 43 | 121 |

## Q7: Would you consider it necessary to request further testing for specific fusion transcripts when EWSR1 break apart is confirmed by FISH?

*Answered: 121 Skipped: 22*

| | NECESSARY | UNNECESSARY | UNSURE | TOTAL |
|---|---|---|---|---|
| Typical Ewing sarcoma morphology, CD99 positive | 29.75% 36 | 61.16% 74 | 9.09% 11 | 121 |
| Atypical Ewing sarcoma morphology or CD99 negative | 76.86% 93 | 12.40% 15 | 10.74% 13 | 121 |

## Q8: Would you consider it necessary to request further testing for specific fusion transcripts when EWSR1 break apart is negative or noninformative?

*Answered: 121 Skipped: 22*

| | NECESSARY | UNNECESSARY | UNSURE | TOTAL |
|---|---|---|---|---|
| Typical Ewing sarcoma morphology, CD99 positive | 80.17% 97 | 7.44% 9 | 12.40% 15 | 121 |
| Atypical Ewing sarcoma morphology or CD99 negative | 84.30% 102 | 4.13% 5 | 11.57% 14 | 121 |

## Q9: What is your discipline?

*Answered: 121 Skipped: 22*

| ANSWER CHOICES | RESPONSES | |
|---|---|---|
| Pediatric oncologist with special interest in sarcoma | 42.15% | 51 |
| Pediatric oncologist without special interest in sarcoma | 33.06% | 40 |
| Surgical pathologist with special interest in sarcoma | 16.53% | 20 |
| Surgical pathologist without special interest in sarcoma | 0.83% | 1 |
| Other (please specify) | 7.44% | 9 |
| TOTAL | | 121 |

## Q10: Where do you currently practice medicine?

*Answered: 121 Skipped: 22*

| ANSWER CHOICES | RESPONSES | |
|---|---|---|
| Africa | 0.00% | 0 |
| Asia | 2.48% | 3 |
| Australia & New Zealand | 1.65% | 2 |
| Europe | 2.48% | 3 |
| North America | 93.39% | 113 |
| South America | 0.00% | 0 |
| TOTAL | | 121 |

**Q11: How many years are you from completion of medical training?**
*Answered: 121 Skipped: 22*

| ANSWER CHOICES | RESPONSES | |
|---|---|---|
| 1 - 5 years | 22.31% | 27 |
| 6 - 10 years | 29.75% | 36 |
| 11 - 15 years | 14.05% | 17 |
| > 15 years | 33.88% | 41 |
| TOTAL | | 121 |

**Q12: What type of practice setting is your institution?**
*Answered: 121 Skipped: 22*

| ANSWER CHOICES | RESPONSES | |
|---|---|---|
| Academic | 90.08% | 109 |
| Private/community | 9.92% | 12 |
| TOTAL | | 121 |

**Q13: Approximately how many newly diagnosed bone and soft tissue sarcoma patients are treated at your institution each year?**
*Answered: 121 Skipped: 22*

| ANSWER CHOICES | RESPONSES | |
|---|---|---|
| 0 - 10 | 29.75% | 36 |
| 11-25 | 28.10% | 34 |
| 26-50 | 16.53% | 20 |
| 51-75 | 5.79% | 7 |
| 76-100 | 1.65% | 2 |
| > 100 | 18.18% | 22 |
| TOTAL | | 121 |

**Q14: Approximately what percentage of your time is spent on basic/translational bench research?**

| ANSWER CHOICES | RESPONSES | |
|---|---|---|
| 0 | 50.41% | 61 |
| 1-20% | 19.01% | 23 |
| 21-40% | 4.96% | 6 |
| 41-60% | 10.74% | 13 |
| 61-80% | 11.57% | 14 |
| 81-100% | 3.31% | 4 |
| TOTAL | | 121 |
